# Psychosoziale Auswirkungen der Pandemie auf Pflegekräfte und Bewohner von Pflegeheimen sowie deren Angehörige – Ein systematisches Review

**DOI:** 10.1007/s00391-021-01859-x

**Published:** 2021-02-23

**Authors:** P. Benzinger, S. Kuru, A. Keilhauer, J. Hoch, P. Prestel, J. M. Bauer, H. W. Wahl

**Affiliations:** 1 Institut für Gesundheit und Generationen, Hochschule für angewandte Wissenschaften Kempten, Bahnhofstraße 61, 87435 Kempten, Deutschland; 2 Geriatrisches Zentrum der Universität Heidelberg, AGAPLESION Bethanien Krankenhaus Heidelberg, Rohrbacher Str. 149, 69126 Heidelberg, Deutschland; 3 Netzwerk Alternsforschung (NAR), Universität Heidelberg, Bergheimer Str. 20, 69115 Heidelberg, Deutschland

**Keywords:** COVID-19, Pandemie, Kontaktbeschränkung, Soziale Isolation, Angehörige, COVID-19, Pandemia, Contact restrictions, Social isolation, Care givers

## Abstract

**Hintergrund:**

Die COVID-19-Pandemie stellt Bewohner von Altenpflegeeinrichtungen, deren Angehörige bzw. Besucher ebenso wie Mitarbeitende vor große Herausforderungen. Viruseindämmende Maßnahmen wirken sich stark auf das Wohlbefinden der betroffenen Personengruppen aus.

**Material und Methode:**

Systematische Literatursuche nach Studien zu psychosozialen Folgen der Pandemie für Bewohner, deren Angehörige bzw. Besucher sowie Mitarbeitende und Zusammenführung der Ergebnisse mittels narrativer Synthese.

**Ergebnisse:**

Es wurden 756 Studien gesichtet, davon 15 Arbeiten eingeschlossen. Die Daten wurden zwischen Februar und Juni 2020 mit Teilnehmenden aus 14 Ländern erhoben. Es wurden v. a. Einsamkeit, Trauer und Depressivität, aber auch Angst, als häufige Reaktionen der Bewohner auf die Kontakt- und Besuchsrestriktionen berichtet. Bewohner mit kognitiven Einschränkungen litten stärker unter den Auswirkungen, auch wenn es gegenteilige Hinweise gibt. Angehörige bzw. Besucher berichteten ebenfalls von einer Zunahme ihrer Einsamkeit und einer reduzierten Lebensqualität. In den Befragungen der Mitarbeitenden schildern diese Angst vor einer Infektion sowohl bei sich als auch bei den Bewohnern. Infizierte Mitarbeitende in den USA äußerten Wut darüber, nicht ausreichend geschützt worden zu sein. Darüber hinaus berichteten Mitarbeitende von einer erheblichen Mehrbelastung.

**Schlussfolgerung:**

Infolge der Pandemie und der ergriffenen Maßnahmen wurden negative psychosozialen Folgen bei Bewohnern, deren Angehörigen bzw. Besuchern und den Mitarbeitenden berichtet. Die abzuleitenden Unterstützungsbedarfe der 3 Personengruppen sind unterschiedlich und sollten bei zukünftigen Maßnahmen hinsichtlich der Pandemie stärker mitevaluiert werden.

**Zusatzmaterial online:**

Die Online-Version dieses Beitrags (10.1007/s00391-021-01859-x) enthält eine Übersicht über die Suchstrategie und eine Übersicht über die Auswahlkriterien und eine Auflistung der eingeschlossenen Studien. Beitrag und Zusatzmaterial stehen Ihnen im elektronischen Volltextarchiv auf https://www.springermedizin.de/zeitschrift-fuer-gerontologie-und-geriatrie/7952776 zur Verfügung. Sie finden das Zusatzmaterial am Beitragsende unter „Supplementary Material“.

## Abstract (en)

**Background:**

Following the COVID-19 pandemic residents of nursing homes, their visitors and families as well as employees are faced with new challenges. Protective measures have a strong impact on the well-being of all these groups of persons.

**Material and methods:**

A systematic search was carried out for studies investigating the psychosocial consequences of the COVID-19 pandemic for residents, their visitors and families as well as nursing home staff. Results were analyzed by narrative synthesis.

**Results:**

A total of 756 studies were screened and 15 studies were included. These studies were conducted between February and June 2020 with participants from 14 countries. Participants reported loneliness, grief and depressive symptoms among residents. Some gave an account of fear as a reaction of residents to social distancing. Residents with cognitive impairment suffered more although there are conflicting reports. The well-being of visitors and friends was compromised and their feeling of loneliness increased. Nursing home personnel reported fear of getting infected and of infecting residents or their own families. Infected workers in the USA expressed anger about a lack of protection. Furthermore, an increase in workload was reported.

**Conclusion:**

Studies conducted during the first months of the pandemic reported negative consequences for the psychosocial well-being of residents, their visitors and nursing home staff. Individual needs for future support of these groups are distinct and need further evaluation during the on-going pandemic.

Ende Januar 2020 wurden die ersten Erkrankungen mit dem neuen Coronavirus SARS-CoV-2 (COVID-19) in Deutschland gemeldet. Durch Infektionsketten zwischen Pflegekräften, Bewohnern und Angehörigen bzw. Besuchern (im Folgenden ausschließlich als „Angehörige“ bezeichnet, aber auf beide Gruppen Bezug nehmend) kam es in Einrichtungen der stationären Altenhilfe zu einer raschen Ausbreitung des SARS-CoV‑2. Analog zum Vorgehen anderer Länder erfolgten ab März 2020 deutliche Zugangs- und Kontaktbeschränkungen in deutschen Pflegeeinrichtungen [26].

Vor diesem Hintergrund wurde ein systematisches Review der verfügbaren internationalen wissenschaftlichen Literatur zu den psychosozialen Auswirkungen der COVID-19-Pandemie auf Pflegeeinrichtungen durchgeführt. Unter psychosozialen Auswirkungen sind in der vorliegenden Übersichtsarbeit Kriterien wie Wohlbefinden, positive und negative Affekte, Ängste, depressive Stimmung und Veränderungen der Kognition gemeint. Der Fokus lag auf Bewohnern, Angehörigen bzw. Besuchern (im Folgenden ausschließlich als „Angehörige“ bezeichnet) sowie Mitarbeitenden.

## Methode

Initial erfolgte im Juni 2020 eine Sichtung der vorhandenen Evidenz; im November 2020 wurde dann eine systematische Literatursuche durchgeführt. Neben 2 Datenbanken (Zusatzmaterial online: Supplement 1: Datenbankrecherchen) erfolgte eine Suche in relevanten Zeitschriften. Einschlusskriterien waren: (1) Studien mit qualitativem oder quantitativem Studiendesign, (2) Teilnehmende waren (a) Bewohner stationärer Pflegeeinrichtungen, (b) deren Angehörige oder (c) Mitarbeitende in stationären Pflegeeinrichtungen, (3) Aussagen zum psychischen Befinden einer der 3 Personengruppen (a–c) seit Beginn der Pandemie, (4) Publikation auf Deutsch oder Englisch. Studien, welche nicht ausschließlich, aber auch die genannten Personengruppen einschlossen, wurden hinsichtlich ihrer Aussagekraft zur Fragestellung geprüft. Die Studienauswahl erfolgte analog des „Preferred Reporting Items for Systematic Reviews and Meta-Analyses (PRISMA) statement“ für systematische Übersichten [23]. Zu Beginn wurde eine Vorauswahl anhand von Titel und Abstract getroffen. Alle verbliebenen Arbeiten wurden im Volltext beschafft und auf die Einschlusskriterien geprüft. Nichteindeutig beurteilbare Studien wurden unabhängig von 2 Autoren bewertet. Aufgrund des Vorliegens von sowohl quantitativen als auch qualitativen Studien wurde eine datenkonvergierende Synthese angestrebt [7]. Entsprechend der strukturellen narrativen Synthese nach Lucas et al. [11] wurden wesentliche Merkmale der Studien (Kontext, Design, Ergebnisse) durch 2 Autoren gemeinsam anhand eines strukturierten Bogens extrahiert (Schritt 1), Hauptbefunde den 3 Subgruppen zugeordnet und eine Zusammenfassung zu jeder Studie verfasst (Schritt 2). Im 3. Schritt erfolgte eine Befundsynthese für jede der 3 Personengruppen. Auf eine Bewertung der Studienqualität wurde aufgrund der methodischen Heterogenität verzichtet. Keine Studie wurde aus Qualitätsgründen ausgeschlossen.

## Ergebnisse

Es wurden 756 Arbeiten identifiziert, von denen 15 in die Synthese eingingen (Zusatzmaterial online: Supplement 2: PRISMA Übersicht; Supplement 3: Übersicht der eingeschlossenen Studien). Insgesamt wurden 7224 Studienteilnehmende aus 14 Ländern berücksichtigt. Drei Studien schlossen Bewohner [3, 12, 28] und 4 Studien Angehörige ein [14, 17, 28, 30], 10 Studien befragten Mitarbeitende [1, 15, 19–22, 24, 25, 28, 29]. Die Stichprobengröße reichte von 24 bis 1997 Teilnehmenden. Die Datenerhebung erfolgte zwischen Februar und Juni 2020. Eine Studie fand während der Lockerung der Restriktionen statt [29]. Der Vergleich mit Daten aus der Zeit vor der Pandemie war in lediglich einer Studie möglich [12]. Die überwiegend gewählte Methode waren Online-Befragungen (10 Studien), die sich etablierter Instrumente, aber auch offener Fragen bedienten. In 4 Studien wurden Interviews geführt.

### Bewohner

In 6 Studien wurde von vermehrter Einsamkeit bei den Bewohnern durch die Besuchsrestriktionen berichtet [17, 20, 24, 25, 28, 30]. Angst als Reaktion der Bewohner auf die Pandemie bzw. die pandemiebedingten Maßnahmen wurde in 2 weiteren Studien beschrieben [15, 28]; auch Traurigkeit und Depressivität wurden erwähnt [24, 25, 28, 30]. Bewohner französischer Pflegeheime gaben für die aktuelle Situation höhere Werte der Subskalen Angst und Depressivität in der Hospital Anxiety and Depression Scale an als für die Zeit vor den Kontaktrestriktionen [3]. Zwei Studien aus den Niederlanden beschrieben eine Zunahme von Unruhe und Aggression bei Bewohnern mit gerontopsychiatrischen Krankheitsbildern [24, 28]. In 3 Studien finden sich Hinweise auf kognitiven Abbau [17, 25, 28], in 2 Studien wurde körperlicher Abbau als Folge der Restriktionen beobachtet [24, 25]. In einer kanadischen Studie wurden in 7 Einrichtungen die Möglichkeit der Videotelefonie für Bewohner eingerichtet und Veränderungen des Wohlbefindens anhand von Routinedaten mit der Situation vor den Besuchsrestriktionen verglichen [12]. So nahm die Häufigkeit eines Delirs, von Depressivität und Verhaltensauffälligkeiten nach Beginn der Restriktionen nicht zu, und die Häufigkeit eines Delirs bei Bewohnern mit Demenz nahm ab [12]. Auch Pflegekräfte aus Deutschland gaben an, dass Bewohner mit Demenz teilweise von der Ruhe in den Einrichtungen profitierten [25].

### Angehörige

Die Auswirkungen der Maßnahmen auf das psychische Befinden von Angehörigen werden in 5 Studien thematisiert [14, 17, 20, 29, 30], wobei die Gruppe in 3 Studien direkt befragt wurde. Dabei zeigte sich ein verringertes psychisches und emotionales Wohlbefinden. So belegt eine irische Studie, dass Angehörige von Bewohnern mit kognitiven Einschränkungen im Vergleich zu Angehörigen von kognitiv gesunden Bewohnern ein deutlich schlechteres Befinden aufwiesen [17]. Jene, die zuvor täglich zu Besuch kamen, zeigten infolge des „social distancing“ ein besonders hohes Maß an Angst und Depressivität. In einer Online-Befragung in den Niederlanden berichteten Angehörige und Freunde von Bewohnern darüber hinaus über Schuldgefühle sowie das Empfinden einer mangelnden Wertschätzung [30]. Eine Studie aus den USA zeigte, dass regelmäßige Telefonate eine emotional zufriedenstellende Kommunikation gewährleisten können [14].

### Mitarbeitende

Vier der 9 Studien, die Mitarbeitende befragten, wurden in Ländern durchgeführt, die in der ersten Welle mit sehr hohen Fallzahlen zu kämpfen hatten. Mitarbeitende erlebten v. a. Angst vor einer Ansteckung sowie Belastungen z. B. durch Einschränkung der Sozialkontakte, Verzicht auf Freizeitaktivitäten sowie Konflikte im professionellen Handeln, etwa den eigenen Pflegestandards weiter zu genügen. Fehlendes Personal, unzureichende Schutzausrüstung [1, 15, 21, 22], häufige Begegnung mit Sterben und Leid sowie aktive COVID-19-Fälle innerhalb der Einrichtung [1, 19] erwiesen sich dabei als Faktoren, die mit einem schlechteren psychischen Befinden assoziiert waren. Infizierte Mitarbeitende in den USA fühlten sich im Stich gelassen und waren wütend über den unzureichenden Schutz [22]. Pflegekräfte aus Italien, Spanien, Mexiko und Peru klagten über eine Stigmatisierung durch ihre Beschäftigung in einer Pflegeeinrichtung [20]. Mitarbeitende in England berichteten auch von Verunsicherungen durch häufig geänderte Hygienevorschriften [15]. Die Aushandlung von Ausnahmen von Besuchsverboten in den Niederlanden stellte für die dort tätigen Ärzte ein frustrierendes Dilemma dar, welches zu Unsicherheit und Schuldgefühlen führte [24]. Vor dem Hintergrund außerordentlicher Belastungen in Norditalien und Spanien sehen 2 Studien das Pandemiegeschehen innerhalb von stationären Altenhilfeeinrichtungen als Ursache einer sekundären Traumatisierung von Mitarbeitenden [1, 19]. Zudem berichten Mitarbeitende über eine erhöhte Arbeitsbelastung [25, 29]. In einer Studie wurde aber auch von einem verbesserten Zusammenhalt innerhalb der Teams, weniger Krankheitsausfällen und einer ruhigeren Arbeitsatmosphäre berichtet [25]. Zwei Studien arbeiteten den Zugang zu sozialer Unterstützung durch Kollegen und Vorgesetzte sowie die Möglichkeit psychologischer Unterstützung als entlastende Faktoren heraus [1, 21].

## Diskussion

Trotz der Heterogenität der Studiendesigns, der national unterschiedlich ausgestalteten Besuchs- und Kontaktrestriktionen sowie der nur bedingt auf die deutsche Situation übertragbaren Befunde verdichtet sich ein Bild, welches die Folgen der Pandemie als emotional belastend für alle untersuchten Gruppen beschreibt.

### Bewohner

Die Beobachtung, dass ältere zu Hause lebende Menschen zu Beginn der Pandemie weniger von psychosozialen Folgen betroffen waren [27], findet keine Entsprechung in der Gruppe der in Altenpflegeeinrichtungen lebenden Menschen. Vielmehr wurde über Einsamkeit sowie weitere psychosoziale Verlusterfahrungen berichtet. Die vorliegenden Befunde beruhen ausschließlich auf den Einschätzungen während der ersten Monate nach Pandemieausbruch, und es ist weiterhin einschränkend zu sagen, dass Einsamkeit bei Bewohnern von Pflegeeinrichtungen bereits vor der Pandemie weit verbreitet war [16]. Es kann kein Zweifel daran bestehen, dass Besuche von Angehörigen ganz wesentlich der Aufrechterhaltung der Wahrnehmung von Kontinuität, Zugehörigkeit und Wertschätzung dienen [9]. Besuchsrestriktionen standen im Gegensatz zur Bedeutung sozialer Interaktion für das Wohlbefinden und zum gesundheitlich-pflegerischen Wohlergehen von Bewohnern. Vor dem Hintergrund von Ergebnissen zu ungünstigen gesundheitlichen Folgen von sozialer Isolation und Einsamkeit bei älteren Menschen [18] verwundern die Befunde aus den Studien nicht. Soziale Kontakte sind auch zur Aufrechterhaltung noch vorhandener kognitiver Reserven von hoher Relevanz und erklären Hinweise auf eine kognitive Verschlechterung von Bewohnern mit Demenz – dies scheint aber auch auf Menschen mit Demenz im ambulanten Pflegekontext zuzutreffen [4]. Interessant vor dem Hintergrund der auch in Deutschland geführten Diskussion zur isolationsmindernden Wirksamkeit von digitalen Kommunikationstechnologien [10] sind die Befunde einer kanadischen Studie [2]. Methodisch nur bedingt schlüssig, unterstützt sie Forderungen in Richtung vermehrter Kommunikationsmedien in Pflegeeinrichtungen. Dies bedarf dringend weiterer empirischer Evidenz.

### Angehörige

Die Not der Angehörigen fand in deutschen Medien ein großes Echo. Dennoch wurde deren Befinden in nur wenigen Studien beleuchtet. Gerade sie verbringen oft viele Stunden pro Woche in den Einrichtungen [6], und ihre Einbindung in die Versorgung kann für sie eine sinnstiftende Aufgabe sein [5]. Die Ergebnisse zeigen deutlich, dass Kontaktbeschränkungen das Belastungserleben von pflegenden Angehörigen verstärken. Einer vertrauensvollen Kommunikation zwischen Einrichtung und Angehörigen, z. B. durch regelmäßige Telefonate, kommt demnach eine wichtige Rolle zu, um so die Sorgen der Angehörigen zu mildern und deren Wohlbefinden zu verbessern [17]. Hierfür müssen jedoch ausreichend personelle Ressourcen in den Einrichtungen zur Verfügung stehen. Lockerungen der Besuchsregelungen werden von Angehörigen begrüßt. Doch müssen Besucher Vertrauen in die Reliabilität und Umsichtigkeit der ergriffenen Hygienekonzepte haben, da fehlendes Vertrauen sie von Besuchen abhalten kann und psychisch weiter belastet [29].

### Mitarbeitende

Die Befunde bestätigen, dass Mitarbeitende in den Einrichtungen in besonderer Weise von den Folgen der Pandemie betroffen waren. Sie sorgten sich um die eigene Gesundheit, die Gesundheit der eigenen Familie und die der Bewohner. Dies geschah vielerorts vor dem Hintergrund einer zeitweise unzureichenden Ausstattung mit persönlicher Schutzausrüstung. Insbesondere in Einrichtungen mit gehäuften Infektionen und Todesfällen erlebten die Beschäftigten die eigene Hilflosigkeit, Kontrollverlust, Schuldempfinden und das erzwungene Zurückbleiben hinter professionellen Standards [25]. Befunde, die eine sekundäre Traumatisierung berichten, sind jedoch vor dem Hintergrund der dramatischen Situation in den Studienregionen (Norditalien, Spanien) vorsichtig zu interpretieren. Bisher sind deutschen Pflegeeinrichtungen solche Zustände weitestgehend erspart geblieben. Dennoch haben auch in Deutschland Mitarbeitende der Altenpflege das höchste Infektionsrisiko aller Berufsgruppen [13, 31] und erleben einen anhaltenden Konflikt in ihrem professionellen Selbstverständnis und Handeln [25]. Sie waren mit einer erhöhten Arbeitsbelastung und einem anspruchsvolleren Arbeitsalltag konfrontiert, was zu einer deutlichen Zunahme von physischer und psychischer Überlastung der Mitarbeitenden führte [8, 25, 26]. In der Folge der Pandemie droht damit eine weitere Verschärfung des Fachkräftemangels in der Altenhilfe. Umso wichtiger ist es, dass die Gesundheit und die Zufriedenheit der Beschäftigten gerade in der Pandemie Beachtung finden. Hinweise auf notwendige Mindestinterventionen ergeben sich auch aus den Befunden des Reviews. So müssen eine adäquate Versorgung mit persönlicher Schutzausrüstung und ein ausreichendes Angebot zur psychosozialen Unterstützung gewährleistet sein. Kollegialer Zusammenhalt sowie Unterstützung durch Vorgesetzte können Stress puffern und zum Erhalt von Arbeitszufriedenheit beitragen. Gerade auch wir Gerontologen und Geriater sind gefordert, Wertschätzung für die geleistete pflegerische Arbeit deutlich hörbar zum Ausdruck zu bringen.

## Fazit für die Praxis

- Eine bessere Versorgung mit Schutzausrüstung sowie das Vorhalten von Antigenschnelltests erlauben inzwischen wieder Besuche in Pflegeeinrichtungen. Solche Maßnahmen können das Sorgen bei Besuchern wie Mitarbeitenden mindern und die Akzeptanz erweiterter Besuchsmöglichkeiten verbessern.
- Auch unter den Bedingungen des „neuen Normals“ muss soziale Teilhabe für Bewohner von Einrichtungen der stationären Altenhilfe gewährleistet sein. Eine entsprechende Leitlinie der Deutschen Gesellschaft für Pflegewissenschaft kann Einrichtungen hier Orientierung verschaffen.
- Der sich rasch verbreiternde Einsatz digitaler Kommunikationsmedien ist erfreulich, muss aber wissenschaftlich begleitet werden. Wie im Achten Altenbericht der Bundesregierung gefordert, sollte WLAN in jedem Bewohnerzimmer die Regel werden. Wo erforderlich, müssen Mitarbeitende qualifiziert werden, Bewohnern den Zugang zu digitalen Kontaktaufnahmen zu ermöglichen und diese dabei zu begleiten.

## Supplementary Information






