# Supplementary material for: Psychosocial effects of the pandemic on staff and residents of nursing homes as well as their relatives—A systematic review
Source: Z Gerontol Geriatr. 2021 Feb 23;54(2):141–5. [Article in German] doi: 10.1007/s00391-021-01859-x (PMC7901511; doi:10.1007/s00391-021-01859-x)
Supplement: Supplementary file 1 [file 391_2021_1859_MOESM1_ESM.pdf]

Supplement 1
Datenbankrecherchen

**Suchstrategie in MEDLINE via Pubmed**

| | Suchbegriffe | Anzahl der Treffer |
|---|---|---|
| #1 | (nursing homes[MeSH Terms]) OR (nursing home[MeSH Terms]) | 667 |
| #2 | "skilled nursing"[Text Word] | 457 |
| #3 | "nursing home"[Text Word] OR "nursing homes"[ [Text Word] | 2.138 |
| #4 | "longterm care"[Text Word] | 5 |
| #5 | "long-term care"[Text Word] OR "long term care"[Text Word] | 1.770 |
| #6 | "elderly home"[Text Word] | 6 |
| #7 | #1 OR #2 OR #3 OR #4 OR #5 OR #6 | 3.794 |
| #8 | ("severe acute respiratory syndrome coronavirus 2"[Supplementary Concept] OR "severe acute respiratory syndrome coronavirus 2"[All Fields] OR "ncov"[All Fields] OR "2019 ncov"[All Fields] OR "covid 19"[All Fields] OR "sars cov 2"[All Fields] OR ("coronavirus"[All Fields] OR "cov"[All Fields]) | 77.709 |
| #9 | #6 AND #7 Filters: German, English | 636 |

Einschränkung: publication date 01/01/2020 – 25/11/2020t, Publikation in Deutsch oder Englisch

**Suchstrategie in CINAHL via EBSCOhost**

| | Suchbegriffe | Anzahl der Treffer |
|---|---|---|
| #1 | TX ( long-term care or nursing home or residential care or assisted living ) OR TX ( skilled nursing facility or nursing home or snf or long term care ) | 4.906 |
| #2 | TX covid-19 or coronavirus or 2019-ncov or sars-cov-2 or cov-19 | 3.198 |
| #3 | #1 AND #2 | 181 |

Einschränkung: peer-reviewed, human, publication date 01/01/2020 – 30/11/2020, English or German
