# Supplementary figures and images for: Psychosocial effects of the pandemic on staff and residents of nursing homes as well as their relatives—A systematic review
Source: Z Gerontol Geriatr. 2021 Feb 23;54(2):141–5. [Article in German] doi: 10.1007/s00391-021-01859-x (PMC7901511; doi:10.1007/s00391-021-01859-x)

Supplement 2  
Preferred Reporting Items for Systematic Reviews and Meta-Analyses

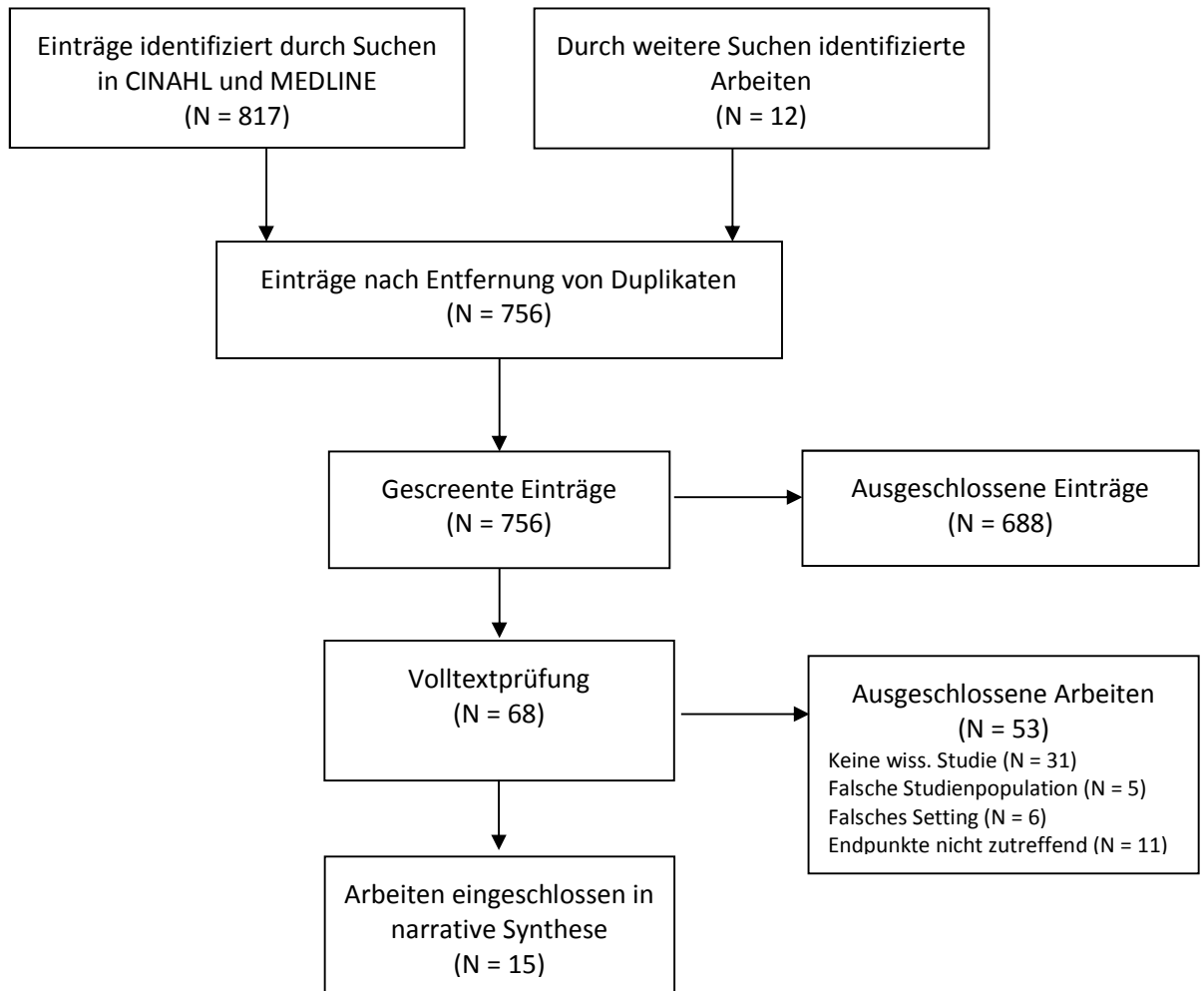

Supplement: Supplementary file 2 [file 391_2021_1859_MOESM2_ESM.pdf]
