# Supplementary material for: Psychosocial effects of the pandemic on staff and residents of nursing homes as well as their relatives—A systematic review
Source: Z Gerontol Geriatr. 2021 Feb 23;54(2):141–5. [Article in German] doi: 10.1007/s00391-021-01859-x (PMC7901511; doi:10.1007/s00391-021-01859-x)
Supplement: Supplementary file 3 [file 391_2021_1859_MOESM3_ESM.pdf]

Supplement 3: Übersicht der eingeschlossenen Studien

Erstautor*in	Land*	Ziel der Studie (wörtliches Zitat)	Daten-erhebung	Erhebungs-instrumente / Themen	Zeitraum	N	Ursprüngliche Stichproben-größe	Rekrutierung	TN	Einschluss-kriterien / Ausschluss-kriterien	Besonderheit
**Blanco-Donso**	ES	"We hypothesized that work stressors would be related to a higher level of secondary traumatic stress and fear of contagion, in contrast to the presence of job resources, which would be negatively related to them. Furthermore,.... we also hypothesize that the presence of sufficient staff and PPEs, as well as coworker and supervisor sup- port in the face of this COVID-19 pandemic, will mitigate the impact of work stressors on secondary traumatic stress and the fear of contagion among professionals working in nursing homes."	Online-Befragung	Hauptendpunkte: Sekundäre Traumatisierung (Subskala des Secondary Traumatic Stress Questionnaire), Angst vor Infektion; weitere Variablen: Arbeitsbelastung (Subskala des Secondary Traumatic Stress Questionnaire), Sozialer Druck durch die Arbeit (Einzelfragen des Secondary Traumatic Stress Questionnaire), Begegnung mit Sterben und Leid (Subskala aus der Nursing Burnout Scale), Unterstützung durch Kollegen und Vorgesetzte (Einzelfragen aus dem Job Content Questionnaire), Versorgung mit PPE	März - April	228	k.A.	Direkte Ansprache von Mitarbeitenden über Soziale Netzwerke (LinkedIn, Twitter), persönliche Kontakte, Weitergabe des Links an Kolleg*innen	**Mitarbeitende**<br>7,4% Ärzt*innen, 19,3% Pflege-fachkräfte, 30,3% Pflege-hilfskräfte, 6,5% "geriatric assistants", 15,3% Sozial-arbeiter*innen, 7,9% Psycho-log*innen, 7,4% Therapieberufe	k.A.	
**El Haj**	FR	"We hypothesized that work stressors would be related to a higher level of secondary traumatic stress and fear of contagion, in contrast to the presence of job resources, which would be negatively related to them. Furthermore,.... we also hypothesize that the presence of sufficient staff and PPEs, as well as coworker and supervisor support in the face of this COVID-19 pandemic, will mitigate the impact of work stressors on secondary traumatic stress and the	Online-Befragung	Primäre Endpunkte: Angst und Depression gemessen mit der Hospital Anxiety and Depression Scale, je zwei verschiedene Instruktionen pro TN: Einschätzung der aktuellen Situation sowie der Situation vor der Einführung von Maßnahmen zur Kontaktreduktion	März-Mai**	58	80, nicht berücksichtigt wurden TN aufgrund einer gemischten Genese der Demenz oder fehlendem MMSE	Direkte Ansprache durch Mitarbeitenden in den Einrichtungen	**Bewohner*innen**	Diagnose einer wahrscheinlichen Alzheimer Demenz, MMSE innerhalb der zurückliegenden drei Monate > 20/30	

		fear of contagion among professionals working in nursing homes."									
**McArther**	CA	"The purpose of our article is to demonstrate how thoughtful use of mitigating strategies (e.g. window visits, use of technology) and clinical information systems like the interRAI LTCF can inform clinical care and prevent worsening mental health outcomes (depression, delirium, and behavioural problems) during the COVID-19 pademic."	Routine-Daten	Primäre Endpunkte: Depression (Depression Rating Scale), Delir (Delirium Clinical Assessment Protocol) und Verhaltens-auffälligkeiten (Aggressive Behaviour Scale) vor den Besuchsrestriktionen und nach Einführung der Restriktionen; weitere Variablen u.a. Soziales Engangement (Revised Index of Social Engagement), Selbständigkeit (Activities of Daily Living Hierarchy), Kognition (Cognitive Performance Scale)	Jan 2017 – Juni 2020	765	k.A.	-	**Bewohner*innen**<br>aus 7 not-for-profit Pflegeeinrichtungen (mittlere Größe 60 Betten)	k.A.	*1*
**Monin**	US	"The objective was to examine what communication methods, other than in-person visits, during the pan-demic were associated with greater positive and lower negative emotional expe- riences for LTC residents and their family members and friends."	Online-Befragung	1) Positive and Negative Affect Scale (PNAS) der Befragten,<br>2) PNAS der Bewohner*innen erhoben über die Einschätzung der Befragten;<br>3) Häufigkeit der Nutzung verschiedener Kommunikations-methoden in der zurückliegenden Woche zwischen Befragten und Bewohnerinnen (Telefonanruf, Telefonanruf während Fensterbesuchs, Videoanruf, Internet/ Telefonchat , Facebookposts, E-Mail, Brief zugestellt durch Mitarbeitende, Briefe zugestellt durch die Post, Abgeben persönlicher Gegenstände (z.B. Fotos).	März – April	161	394, Aus-schlüsse nach Plausibilitäts-prüfung (N = 29), Angehöriger / Freund lebt nicht in einer Pflegeeinricht-ung (N = 1), Angehöriger / Freund lebt nicht in den USA (N = 7), TN lebt nicht in den USA (N = 91), Bewohner*in < 55 Jahre	gezielte Emails, Social Media Posts, Amazon's Mechanical Turk	**Angehörige / Besucher**	Angehöriger oder nahestehende_r Freund*in, der / die in einer stationären Pflege-einrichtung lebt; mindestens 1 x Monat Kontakt vor den Besuchseinschränkungen	
**Nyashahu**	UK	"This paper was set out to explore the challenges faced by different frontline workers in health	Interviews via Video-telefonie	Explorativ	Feb. - April	40	k.A.	30 Pflegeanbieter (stationäre Pflegeeinrichtungen, ambulante	**Mitarbeitende**<br>(N=40) von 20 Pflegeanbietern (stationäre (N = 15) und häusliche	k.A.	

		and social care during the COVID-19 pandemic."						Pflegedienste) wurden kontaktiert	Pflege (N=15) aus den Berufsgruppen Pflege (N = 15), Verwaltung (N = 10) und Hilfskräfte (N = 15)		
**O'Caoimh**	IE	"We examined the effects of COVID-19 visiting restrictions on measures of perceived loneliness, well-being, and carer quality of life (QoL) amongst visitors of residents with and without cognitive impairment (CI) in Irish RCFs."	Online-Befragung	Selbstauskunft der Bezugsperson über die Zufriedenheit mit der Pflege, den Einfluss der Restriktionen auf die Kommunikation mit der Einrichtung, Einsamkeitserleben (University of California Los Angeles brief loneliness scale), psychologisches Wohlbefinden (World Health Organization Five Well-being Index - WHO-5), Lebensqualität (Adult Carer Quality of Life Questionnaire (AC-QoL) stratifiziert nach CI des / der Bewohner*in	Juni	202	230<br>Ausschlüsse nach Plausibilitätsprüfung (N = 5), fehlende Angaben zu CI (N = 23)	Email-Listen, Social media Accounts von Lokalzeitungen	**Besucher*innen**<br>91% Familien-angehörige	Bewohner*in lebten in Irland	
**Riello**	IT	"In this survey-based epidemiological study, we test the prevalence of anxiety and post-traumatic symptomatology in residential nursing and care home workers—a group of individuals that has been largely neglected but who nonetheless plays a very important and sensitive role in our society. We do this by focusing on the North of Italy, the most affected region during the first COVID-19 outbreak in Italy."	Online-Befragung	Symptome einer Posttrauma-tischen Belastungsreaktion (Impact of Event Scale Revised (IES-R)), Angststörung (Generalized Anxiety Disorder-7 (GAD-7)	Juni - Juli	1071	1140,<br>Ausschlüsse nach Plausibilitäts-prüfung / Prüfung der Vollständigkeit (N = 61) und Tätigkeit: nicht kategorisier-bar (N = 4), nicht in der Einrichtung (N = 4)	188 Einrichtungen wurden per Email kontaktiert, 33 nahmen an der Studie teil	**Mitarbeitende**<br>75,6% health care staff, 13,6% technical staff, 18,8% Verwaltung	Einrichtungen in Norditalien	
**Sarabia-Cobo**	ES, IT, PE, MX	"The purpose of this study was to use in-depth interviews to explore the emotional impact and experiences of registered nurses working in nursing homes facing extraordinary epidemic situations during these	Halb-strukturierte Interviews via Video-telefonie	Interviewleitfaden mit den Themen: 1.) Ängste im Zusammenhang mit der pandemischen Situation, 2.) Pflichtgefühl und commitment to care und 3.) emotionale Erschöpfung	April	24	k.A.	gezielte Stichproben-gewinnung von Schlüssel-informanten aus Einrichtungen des gleichen Trägers	**Mitarbeitende**<br>Pflegefachkräfte (Registered Nurses) (7 aus Spanien, 7 aus Italien, 4 aus Peru, 6 aus Mexiko) aus 14 Einrichtungen	Einschlusskriterien: Infizierte Bewohner*innen / Mitarbeitende in der Einrichtung, Tätigkeit in der Einrichtung mind. 6 Monate, Spanische Sprache	

		months, to provide a perspective for designing interventions focused on emotional impact management."									
**Senczyszyn**	PL	"Therefore, the aim of this study was to assess psychological conse- quences (somatic symptoms, anxiety and insomnia, social dysfunc- tion, and depression) among LTCF employees exposed to the SARS-CoV-2 coronavirus pandemic crisis. In addition, we investigated if factors such as PPE availability, safety guidelines, or access to psy- chiatric and psychological support at the workplace correlated."	Online-Befragung	Einschätzung der Veränder-ungen bei Bewohner*in (Stimmung, Kognition, Selbständigkeit) durch Bezugsperson. Einschätzung zum Umgang des / der Bewohner*in mit den Restriktionen (bspw. auf Grundlage von Interaktionen oder Telefongesprächen)	Mai - Juni	178	Die Umfrage wurde 242 Mal aufgerufen, 12 TN wurden ausgeschlossen, da > 70% der Fragen unbeantwortet waren.	k.A.	**Mitarbeitende**<br>keine Angaben zu dem Anteil der jeweiligen Berufs-gruppen an der Befragung	k.A.	
**Seshadri**	US	"Herein, we report on nursing home HCW experiences, and we recommend measures to better support them."	Interviews telefonisch geführt	k.A.	Mai - Juni*	161	k.A.	k.A.	**Mitarbeitende**<br>Gesundheitsfach-kräfte aus 28 Pflegeeinrichtungen	Einschlusskriterium: positiver Test auf eine Infektion mit dem SARS-CoV-2 Virus.	
**Sizoo**	NL	"The aim of this study was an exploration of dilemmas experienced by elderly care physicians (ECPs) as a result of the 73 COVID-19 driven restrictive visiting policy."	Online-Befragung	Offenen Fragen zu erlebten Dilemmas und schwierigen Situationen im Context der Pandemie	April - Mai	76	76	Emails an Weiterbildungs-einrichtungen und Weiterbilder*innen sowie Ärzt*innen in der Geriatrie-Weiterbildung, Möglichkeit zur Weiterleitung an Kolleg*innen	**Mitarbeitende**<br>Geriater*innen (Elderly Care Physicians) und Ärzt*innen in der Geriatrie-Weiter-bildung, die Pflege-heimbewohner *innen betreuen	k.A.	

**Sporket**	DE	"Befragt wurden Mitarbeitende aller Berufsgruppen und Bereiche zu der Frage, wie sie die Situation in den Einrichtungen für die Bewohnerinnen und Bewohner wahrnehmen und einschätzen und wie sich die Arbeit in der Einrichtung unter den gegebenen Bedingungen gestaltet."	Online-Befragung	Offene Fragen zur Einschätzung der Auswirkungen der corona-bedingten Restriktionen im Pflegeheim auf Bewohner und Wahrnehmung der Arbeits-situation für Mitarbeitende<br>Themen:<br>1.) Einschätzung des Besuchs-verbots und weiterer Problem-felder<br>2.) Wahrnehmung und Umgang mit der Situation seitens der Bewohner*innen<br>3.) Auswirkungen auf an Demenz Erkrankte bzw. kognitiv beeinträchtigte Menschen<br>4.) Einschätzung der Arbeitssituation bzw. -belastung<br>5.) Gewünschte Unterstützung<br>6.) Positive Aspekte	April – Mai	34	k.A.	Mitarbeitende von vier Einrichtungen eines Trägers, k.A. zur Ansprache / Rekrutierung	**Mitarbeitende**<br>20 Pflegekräfte, 7 Betreuungskräfte, 1 Hauswirtschaftskraft, 6 Leitungskräfte	k.A.	
**van der Roest**	NL	"The study aims to gain insight into the consequences of the COVID-19 measures on loneliness, mood, behavioral problems in residents in Dutch LTCFs."	Online-Survey	*Bewohner*innen*: Stimmung (Mental Health Inventory 5-index), Änderung in der Stimmung seit Beginn der Besuchsrestriktionen;	April – Mai	193	k.A.	Einladung wurde an 357 Einrichtungen gesendet mit Bitte um Weiterleitung	**Bewohner*innen**<br>stationärer Pflege-einrichtungen und Betreutem Wohnen	Einschlusskriterium: keine schwere CI	
				Angehörige: Änderung in der Stimmung der Bewohner*innen;		623	811,<br>Ausschluss, wenn nicht unmittelbar an Pflege beteiligt		**Mitarbeitende**<br>Gesundheits-fachberufe	k.A.	
				Gesundheitsfachkräfte: Änderungen in der Ausprägung neuropsychiatrischer Symptome / Verhaltensauffälligkeiten (pro Wohneinheit) anhand von 10 Domänen des Neuro-psychiatrischen Inventars;		1387	1609,<br>Ausschluss wenn in den 4 Wochen vor Beginn der Besuchs-restriktionen kein Kontakt		**Angehörige**	k.A.	
				Einsamkeit (international etablierte 1-Item-Messung) bewertet durch alle drei Gruppen							

**Verbeek**	NL	"This study reports the first findings on how the national guideline was applied in the local context of the nursing homes, the compliance to local protocols, and the impact on well-being of residents, their family caregivers, and staff. "	Mixed-method Design: Online Frage-bogen, Telefoninter views, Dokumente n-analysen, Datenbank-analyse zu Infektionsza hlen, Auswertung des Chats einer WhatsApp Gruppe	Themen der Befragungen und Auswertungen: 1.) Umsetzung der Vorgaben in den jeweiligen Einrichtungen, 2.) Erste Erfahrungen seit Lockerung der Besuchs-restriktionen, 3.) Hürden und begünstigende Faktoren bei der Umsetzung der Vorgaben, 4.) Einschätzung der Aus-wirkungen auf psychisches Wohlbefinden von Bewohnern, Besuchern und Mitarbeitenden	Mai - Juni	26	26 Einrichtungen	Je eine Person aus jeder Pflege-einrichtungen	**Mitarbeitende** keine Angaben zur Profession oder Rolle in den Einrichtungen	-	*2*
**Wammes**	NL	"The aim of this study was to capture perspectives from the relatives of nursing home residents on nursing home visiting restrictions. Design: A cross-sectional online survey was conducted."	Online-Befragung	Offenen und geschlossenen Fragen zu folgenden Aspekten: 1.) Zugang zu Kommuni-kationsmöglichkeiten, 2.) mögliche negative Auswirkungen der Besuchsrestriktionen auf Bewohner*innen und deren Angehörige, 3.) mögliche schützende Auswirkungen der Besuchs-restriktionen, 4.) für Angehörige wichtige Aspekte während und nach dem Ende der Besuchs-restriktionen	April - Mai	1997	3316 Interessierte; 1117 ausge-schlossen, da die Einschluss-kriterien nicht erfüllt wurden, 202 ausge-schlossen, da keine Frage beantwortet	Webseiten einer Seniorenorganisation, einer Pflege-Gewerkschaft, Selbsthilfeorganisation und zwei nationale Zeitungen	**Angehörige**	Einschlusskriterien: Bewohner*in ist Familienangehörige_r, Bewohner*in lebt während der Zeit der Besuchsrestriktion in einem Pflegeheim , TN mind. 18 Jahre alt, ausreichende Sprachkenntnisse	

* Herkunftsland der Teilnehmenden, ** nach persönlicher Rücksprache mit dem Autor;

1: Studie mit pre-post Daten: nach Beginn der Kontaktbeschränkungen wurde Personal gezielt einsetzte, um den Kontakt zwischen Bewohner*innen und Angehörigen sicherzustellen, es wurde 1 iPad pro 10 Bewohner*innen für Videotelefonie zur Verfügung gestellt, eine der Einrichtungen hatte Studentische Hilfskräfte, welche Besuche durchführten und bei Videotelefonie behilflich waren.

2: In den 26 von den Gesundheitsbehörden ausgewählten Einrichtungen wurde eine Lockerung der Besuchsrestriktionen unter Einhaltung von nationalen Vorgaben erlaubt.

CI: Cognitive Impairment (Kognitive Einschränkung), HCW: Health Care Worker (Gesundheitsfachkräfte), LTCF: Long Term Care Facility (Pflegeheim), k.A.: keine Angaben, N: Anzahl, PPE: Personal Protective Equipment (Persönliche Schutzausrüstung), RCF: residential care facility (Pflegeheim), TN: Teilnehmende
